# Supplementary material for: Anti-Osteoporotic Effects of Kukoamine B Isolated from Lycii Radicis Cortex Extract on Osteoblast and Osteoclast Cells and Ovariectomized Osteoporosis Model Mice
Source: Int J Mol Sci. 2019 Jun 6;20(11):2784. doi: 10.3390/ijms20112784 (PMC6600412; doi:10.3390/ijms20112784)
Supplement: Supplementary file 1 [file ijms-20-02784-s001.pdf]

Anti-Osteoporotic Effects of Kukoamine B Isolated from *Lycii Radicis* Cortex Extract on Osteoblast and Osteoclast Cells and Ovariectomized Osteoporosis Model Mice

Eunkuk Park, Jeonghyun Kim, Mun-Chang Kim, Subin Yeo, Jieun Kim, Seulbi Park, Miran Jo, Chun Whan Choi, Hyun-Seok Jin, Sang Woo Lee, Wan Yi Li, Ji-Won Lee, Jin-Hyok Park, Dam Huh and Seon-Yong Jeong

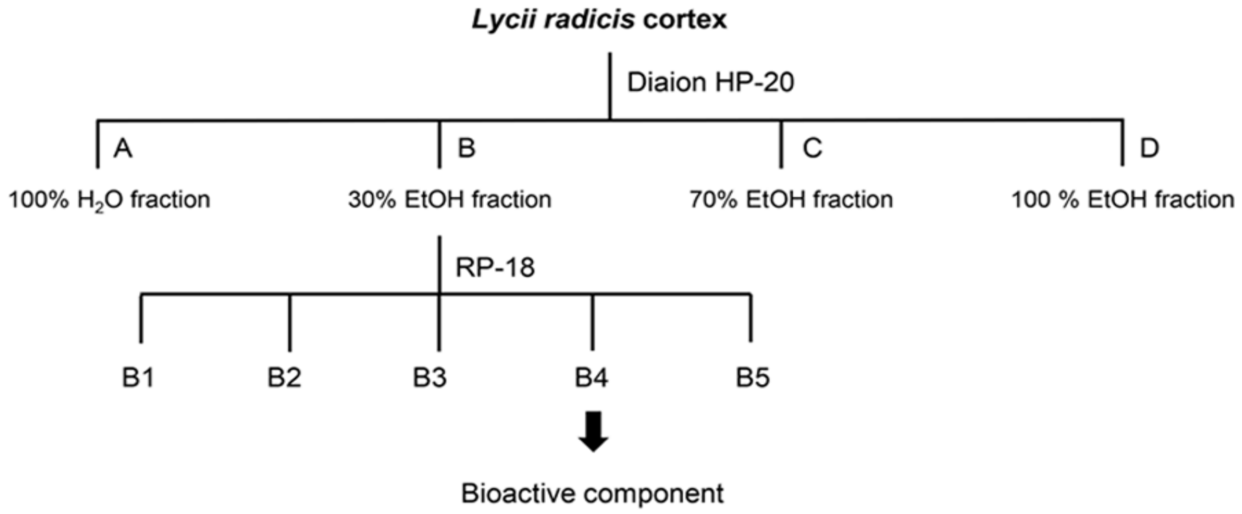

Figure S1. Fractionation and isolation of the bioactive component enhancing osteoblast differentiation from 30% ethanol extract of *Lycii radicis* cortex.

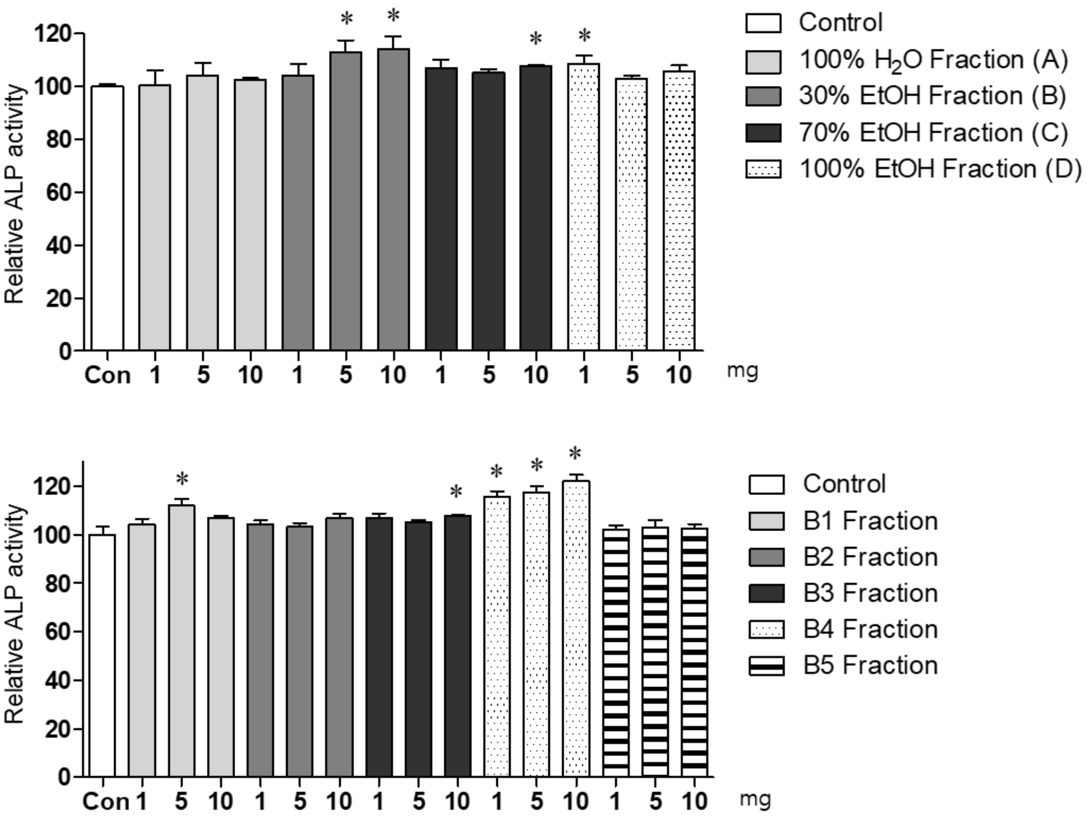

Figure S2. ALP activity test of the fractions isolated in Figure S1 using the pre-osteoblast MC3T3-E1 cells. Cells were treated with ascorbic acid (50 μg/ml) and β-glycerophosphate (10 mM) and cultured with three different concentrations (1, 5, and 10 mg), and an ALP activity was assessed. Control: non-treated cells. *: $p < 0.05$ vs. control.

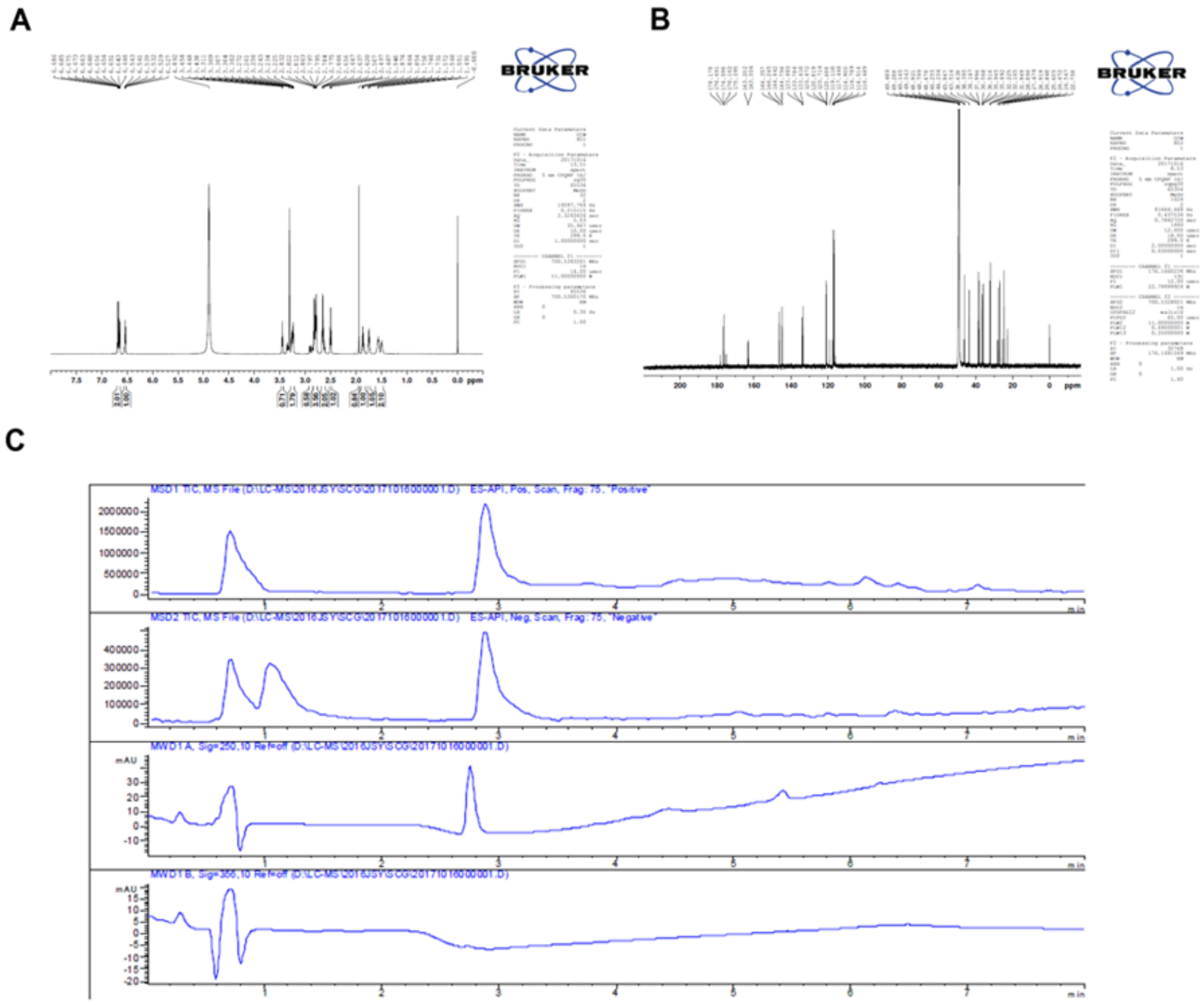

Figure S3. Results of proton nuclear magnetic resonance (^{1}H-NMR). (A) Carbon-13 nuclear magnetic resonance (^{13}C-NMR), (B) mass spectrum, and (C) analyses of the B4 fraction of Supplementary Fig. S1.

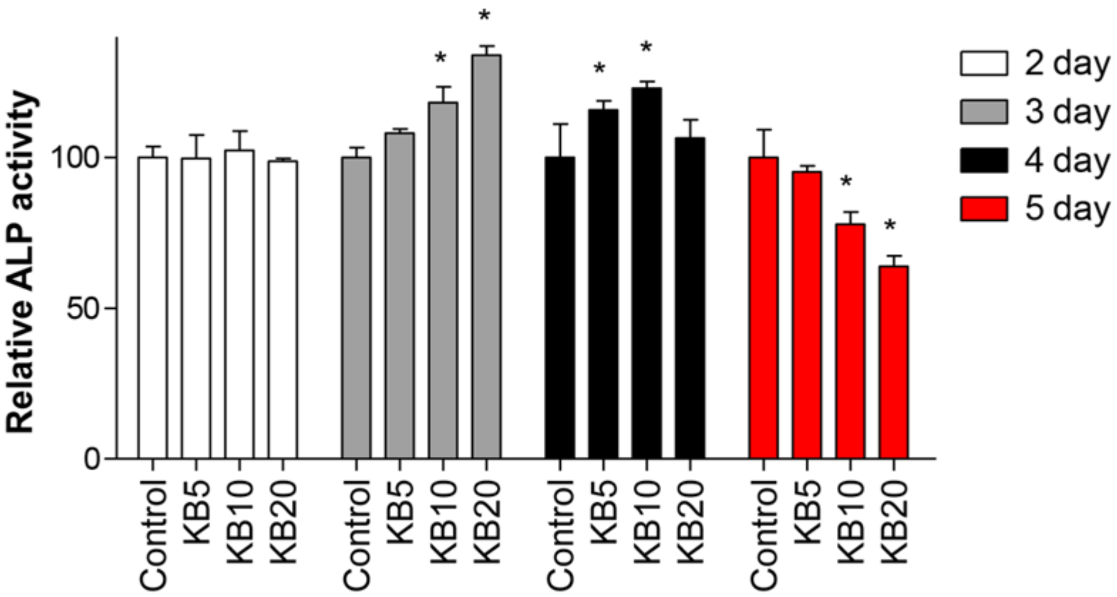

Figure S4. Effects of kukoamine B (KB) on alkaline phosphatase (ALP) activity in preosteoblast MC3T3-E1 cells. Cells were treated with ascorbic acid (50 μg/ml) and β-glycerophosphate (10 mM) and cultured with three different concentrations of KB (5, 10, and 20 μM), and an ALP activity assay was done at 2, 3, 4, and 5 days of incubation. Control: KB non-treated cells. *: $p < 0.05$ vs. control.

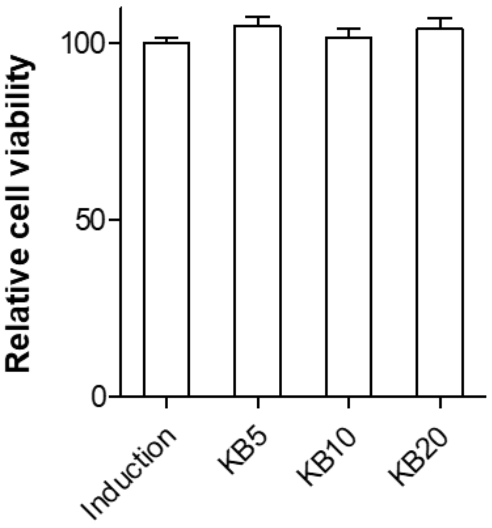

Figure S5. Effects of kukoamine B (KB) on cell viability of primary-cultured monocytes. After induction of osteoclast differentiation by treatment of 30 ng/ml of M-CSF and 50 ng/ml of RANKL (Induction), cells were co-treated with three different concentrations of KB (5, 10, and 20 μM) for 6 days, and then cell viability was assessed.

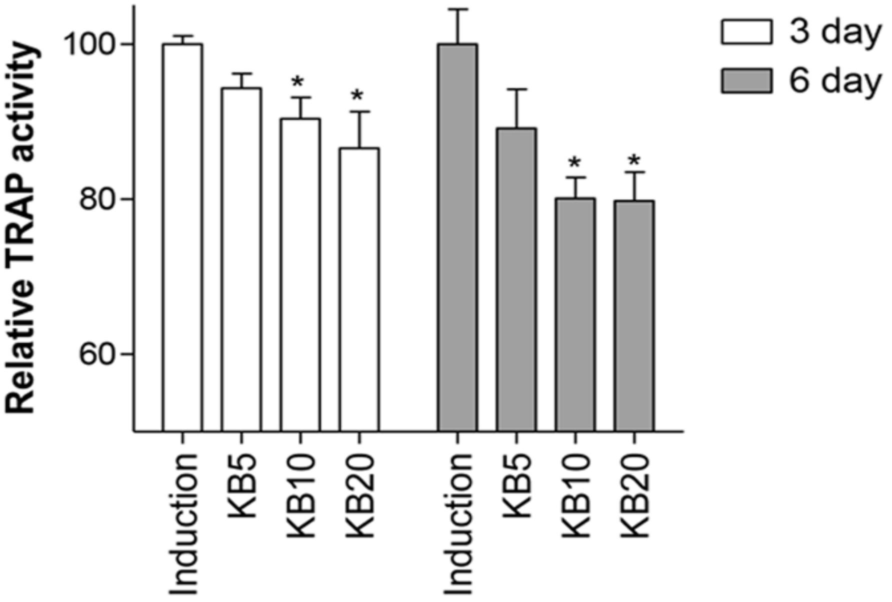

Figure S6. Effects of kukoamine B (KB) on osteoclast differentiation of primary-cultured monocytes. After induction of osteoclast differentiation by treatment of 30 ng/ml of M-CSF and 50 ng/ml of RANKL (Induction), cells were co-treated with three different concentrations of KB (5, 10, and 20 μM) for 3 and 6 days, and tartrate-resistant acid phosphatase (TRAP) activity was assessed. *: $p < 0.05$ vs. Induction.

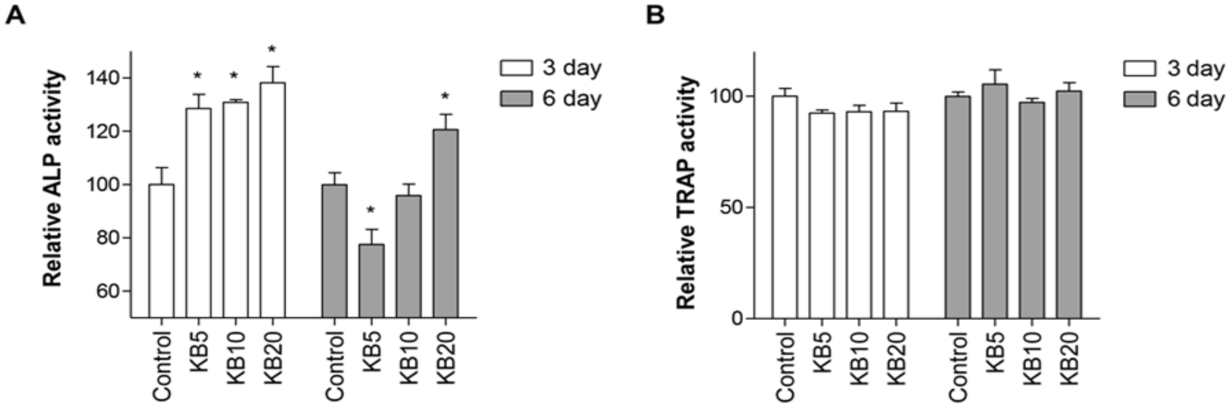

Figure S7. Effects of kukoamine B (KB) on osteoblast and osteoclast differentiations in the co-culture of pre-osteoblasts and primary monocytes. Co-cultured MC3T3-E1 and primary monocyte cells were treated with osteoblast differentiation reagents, 50 μg/mL of ascorbic acid, and 10 mM of β-glycerophosphate and then co-treated with three different concentrations of KB (5, 10, and 20 μM) for 3 and 6 days. Alkaline phosphatase (ALP) activity (A) and tartrate-resistant acid phosphatase (TRAP) activity (B) were assessed in the co-culture cells. Control: KB non-treated cells. *: $p < 0.05$ vs. Control.
